# *O*-GlcNAc Dynamics: The Sweet Side of Protein Trafficking Regulation in Mammalian Cells

**DOI:** 10.3390/cells12101396

**Published:** 2023-05-15

**Authors:** Awatef Ben Ahmed, Quentin Lemaire, Jodie Scache, Christophe Mariller, Tony Lefebvre, Anne-Sophie Vercoutter-Edouart

**Affiliations:** Univ. Lille, CNRS, UMR 8576—UGSF—Unité de Glycobiologie Structurale et Fonctionnelle, F-59000 Lille, France

**Keywords:** *O-*GlcNAcylation, trafficking, secretory pathway, Golgi apparatus, endosome, exocytosis, endocytosis, clathrin, extracellular vesicles

## Abstract

The transport of proteins between the different cellular compartments and the cell surface is governed by the secretory pathway. Alternatively, unconventional secretion pathways have been described in mammalian cells, especially through multivesicular bodies and exosomes. These highly sophisticated biological processes rely on a wide variety of signaling and regulatory proteins that act sequentially and in a well-orchestrated manner to ensure the proper delivery of cargoes to their final destination. By modifying numerous proteins involved in the regulation of vesicular trafficking, post-translational modifications (PTMs) participate in the tight regulation of cargo transport in response to extracellular stimuli such as nutrient availability and stress. Among the PTMs, *O*-GlcNAcylation is the reversible addition of a single N-acetylglucosamine monosaccharide (GlcNAc) on serine or threonine residues of cytosolic, nuclear, and mitochondrial proteins. *O*-GlcNAc cycling is mediated by a single couple of enzymes: the *O*-GlcNAc transferase (OGT) which catalyzes the addition of *O*-GlcNAc onto proteins, and the *O*-GlcNAcase (OGA) which hydrolyses it. Here, we review the current knowledge on the emerging role of *O*-GlcNAc modification in the regulation of protein trafficking in mammalian cells, in classical and unconventional secretory pathways.

## 1. Introduction

**Figure 1:**
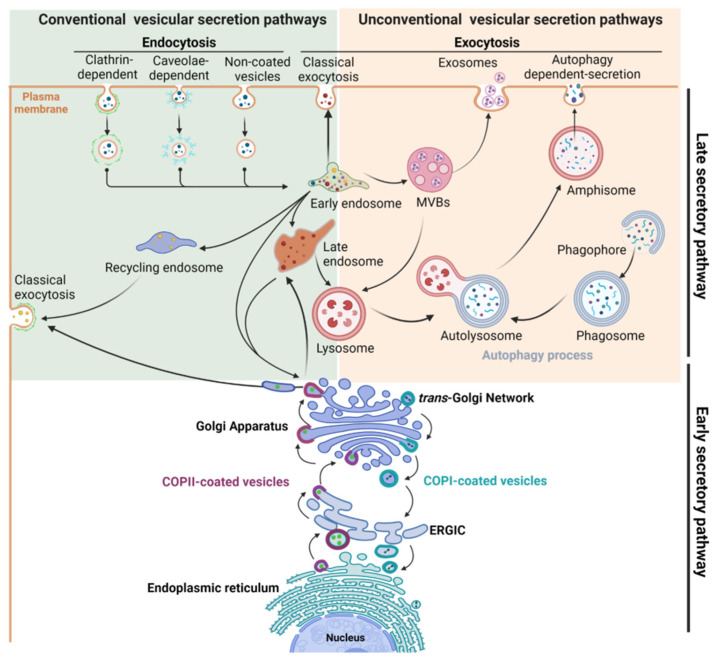
Schematic view of the intracellular compartments and organelles involved in the main conventional and unconventional secretory pathways.

Transport of proteins between the cellular compartments and the cell surface is governed by intricate molecular networks called the secretory pathway. Intracellular trafficking of newly synthesized proteins occurs between the endoplasmic reticulum (ER) to the Golgi apparatus, allowing the processing and maturation of glycoproteins, the targeting of proteins to their final compartment, or the recycling of macromolecules. This is supported by the early secretory pathway that encompasses the anterograde transport of cargoes from the ER to the Golgi, and conversely, their retrograde transport from the Golgi to the ER [1,2,3]. Then, the late secretory pathway takes over to transport mature proteins to their final destination, i.e., the plasma membrane, the extracellular space or organelles of the endolysosomal compartment [4,5] (Figure 1). This implies both the constitutive and regulated secretory pathways which are important for maintaining cellular homeostasis and exocytosis of specific cargoes in differentiated cells, respectively [6]. Conversely, plasma membrane proteins are internalized by endocytosis through clathrin-dependent or clathrin-independent mechanisms. Endocytosed cargoes are usually transported into early endosomes to be sorted and sent to late endosomes and lysosomes for degradation, to the trans-Golgi network (TGN), or to the recycling endosomes to be routed back to the cell surface [4]. Finally, proteins can bypass the Golgi to reach cell surface using unconventional secretion pathways. Three types of organelles are involved in the unconventional secretion pathways that are mostly induced by stress: multivesicular bodies (MVBs) [7], autophagosomes and lysosomes [8] (Figure 1).

**Figure 2:**
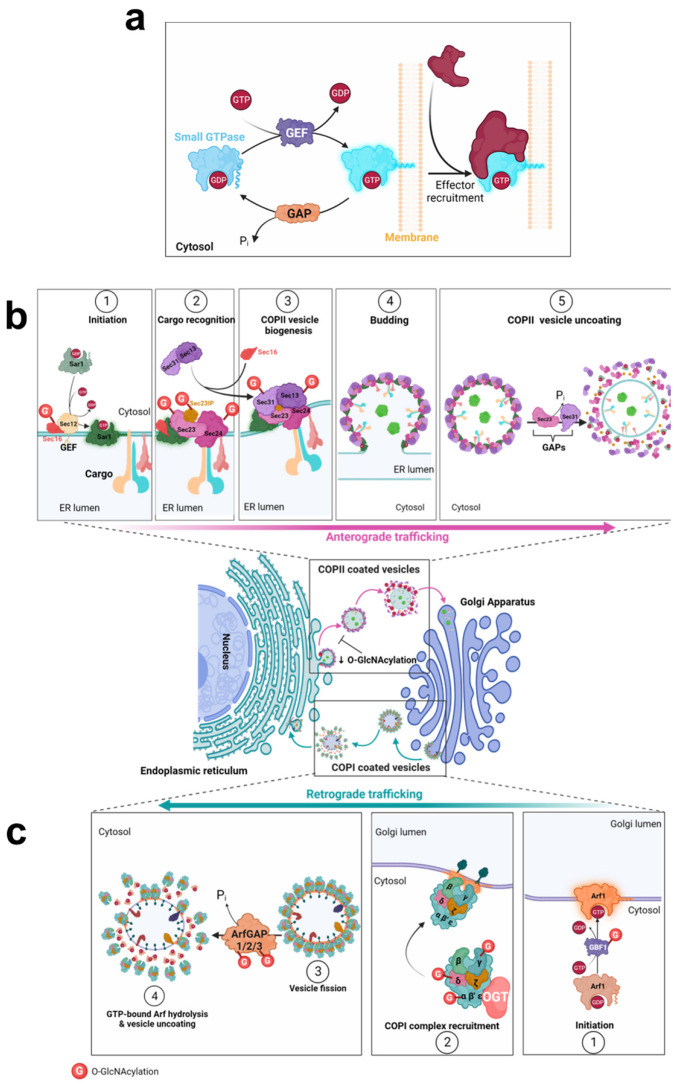
*O*-GlcNAcylation of COPII and COPI trafficking machineries. *O*-GlcNAc modification (G) is indicated by a red circle on the target proteins of OGT. (**a**), Initiation of the assembly or disassembly of COP complexes is mediated by small GTPase proteins, according to their GTP or GDP state. (**b**), Anterograde trafficking of COPII vesicles. (**c**), The retrograde transport of ER- and Golgi-resident proteins is mediated by COPI-coated vesicles.

The secretory pathway relies on a myriad of signaling and regulatory proteins involved in the formation, budding, transport, or fusion of vesicles with the targeted membrane. They act sequentially and in a well-orchestrated manner to ensure the proper delivery of cargoes to their final destination. Among these key molecular actors, specific proteins form the vesicular coat, such as the coat protein complexes (COP) involved in transport between the ER and the Golgi, and the clathrin chains, the major components of clathrin-coated vesicles (CCVs) involved in the exocytic and the endocytic traffics. Beside these structuring proteins, many adaptor and accessory proteins are required, such as Adaptor Protein (AP) complexes that are crucial for sorting of cargo from TGN and for internalization of cell surface cargoes [9]. Other crucial regulatory factors are the small GTPase proteins, including ADP-ribosylation factor (Arf) and Rab proteins, their effectors and regulators (Figure 2a), which are involved in many steps from the formation of transport vesicle at the donor compartment to its fusion at the target membrane [9,10,11,12]. The final steps of vesicle docking and fusion are controlled by soluble NSF attachment protein receptor (SNARE) complexes. SNARE proteins that are localized in opposing membranes ensure the delivery of cargoes to their appropriate place by driving membrane fusion [13].

Post-translational modifications (PTMs) participate in the regulation of trafficking by modifying protein–protein interaction, complex assembly and cargo transport. PTMs not only occur on accessory and regulatory proteins, but also on protein cargoes themselves. For example, ubiquitination acts as a signal for the internalization and sorting of plasma membrane proteins such as tyrosine kinase receptors and nutrient transporters [14,15,16]. Numerous kinases such as ERK, Akt, AMPK and Src, are activated by external stimuli and propagate signaling cascades by phosphorylating specific targets including trafficking-related proteins [17,18,19,20,21,22]. Furthermore, kinases associated with the trafficking machinery act directly or indirectly on key players of the secretory pathway, such as adaptor-associated kinase 1 (AAK1), which is activated by clathrin assembly [23], and protein kinase D (PKD) at the TGN that regulates the post-Golgi transport of E-cadherin [24,25]. In addition to these well-studied PTMs, there is increasing evidence that *O*-GlcNAcylation plays a role in the trafficking of proteins in the secretory pathways.

*O*-GlcNAcylation is the reversible addition of a single *O*-linked-β-*N*-acetylglucosamine monosaccharide (*O*-GlcNAc) on a wide range of cytoplasmic, nuclear and mitochondrial proteins. In contrast to ubiquitination and phosphorylation cycles that are carried out by a myriad of ubiquitin ligases/deubiquitinases and kinases/phosphatases, respectively, the *O*-GlcNAc cycle is controlled by a unique pair of enzymes which are ubiquitously expressed: The *O*-GlcNAc transferase (OGT) and *O*-GlcNAcase (OGA). OGT adds the GlcNAc moiety on the hydroxyl group of Ser/Thr (S/T) residues using the nucleotide-sugar UDP-GlcNAc as a donor substrate, whereas OGA removes the *O*-GlcNAc moiety [26,27].

UDP-GlcNAc is the final product of the hexosamine biosynthetic pathway (HBP) which depends on major metabolic pathways including carbohydrates, fatty acids and amino acids metabolisms. OGT is therefore seen as a sentinel of nutrient availability, helping cells to adapt their biological response to changes in the extracellular environment [28,29,30]. In addition, *O*-GlcNAcylation can occur co-translationally on nascent proteins, as evidenced for a set of proteins including Sp1 and Nup62 [31]. Moreover, a non-catalytic function of OGT is required for normal cellular growth, highlighting an additional role of OGT on its protein partners independently of its glycosyltransferase activity [32].

The full-length isoforms of OGT and OGA are abundantly expressed in the cytoplasm and nucleus, where they control the *O*-GlcNAc cycle on a wide range of proteins, including signaling proteins, cytoskeletal and structural proteins, ribosomal proteins and transcription factors [29,33]. *O*-GlcNAc modification regulates crucial protein functions, such as cellular localization, stabilization, and bioactivity of protein targets. *O*-GlcNAc PTM also modifies protein–protein interactions, hence regulating interactions in oligomeric enzymes [34,35] and the assembly of multiprotein complexes [36,37,38]. Moreover, *O*-GlcNAcylation can reduce the propensity of proteins to aggregate [39,40]. In addition, a potential competition between *O*-GlcNAc and other PTMs, such as ubiquitination and phosphorylation, may occur at the same or adjacent residues, adding to the complexity of understanding the direct molecular functions of *O*-GlcNAc PTM [29,33,41,42,43,44,45,46]. For example, alternative modification of c-Myc at T58 by phosphorylation or *O*-GlcNAcylation differentially regulates its functions, in particular its proteasomal degradation [47]. For the catalytic subunit of casein kinase 2, CK2α, *O*-GlcNAcylation at S347 inhibits phosphorylation at a proximal site, T344. This affects the phosphorylation-dependent stabilization of CK2α. Furthermore, reciprocal phosphorylation and *O*-GlcNAc modifications at these proximal sites significantly alter the protein substrate selectivity of CK2, without modulating its catalytic efficiency with peptide substrates [48]. Conversely, phosphorylation of OGT can regulate its glycosyltransferase activity. For example, phosphorylation at T444 of OGT by AMP-activated protein kinase (AMPK) alters its subcellular localization and substrate selectivity [49]. More recently, EGF-induced tyrosine-phosphorylation of OGT at Y976 was shown to promote its association with pyruvate kinase M2 (PKM2). This induces an upregulation of PKM2 *O*-GlcNAcylation, resulting in destabilization of PKM2 tetramers and hence a reduction in PKM2 activity [35].

Given the broad range of OGT’s targets, *O*-GlcNAc cycling plays critical role in numerous cellular processes, such as signaling pathways, stress response, transcriptional and epigenetic regulation [29,50,51,52,53]. A role for *O*-GlcNAc has also emerged in the regulation of trafficking and secretion of proteins. Notably, *O*-GlcNAc cycling regulates the secretion of cytokines to modulate inflammatory responses, mainly through transcriptional-dependent mechanisms. Readers are referred to recent studies and comprehensive reviews for a more detailed overview of this topic [54,55,56,57]. Here, we review the evidence characterizing the role of *O*-GlcNAcylation on components of the molecular trafficking machinery in the early and late secretory. We also analyze the effects of *O*-GlcNAc dynamics on the modulation of trafficking and bioactivity of various cargoes. Finally, we discuss the current knowledge on *O*-GlcNAcylation and unconventional secretion pathways.

## 2. Overview of *O*-GlcNAcylated Proteins Involved in the Regulation of Vesicle Trafficking

Thanks to methodological developments and mass spectrometry analyses, a considerable number of proteins involved in trafficking have been identified as OGT substrates. Based on the two *O*-GlcNAcome databases recently implemented, oglcnac.mcw.edu (v1.3) [58] and oglcnac.org (O-GlcNAcAtlas_2.0) [59], we selected the *O*-GlcNAcylated proteins involved in the secretion machinery and classified them according to the pathway they are involved in: the early secretory pathway (Table 1), the clathrin-dependent or -independent endocytosis (Table 2) and the unconventional secretory pathways (Table 3). Original publications on the experimental identification of *O*-GlcNAc sites and proteins can be found in the databases. Despite the fact that *O*-GlcNAc sites have been identified on many of them, little is known about their specific function. In the following sections, we will describe more particularly the current knowledge on the role of *O*-GlcNAc cycling on the regulation of these pathways.

## 3. COPII and COPI Machineries

Transport across the early secretory pathway is crucial to ensure the proper PTMs and processing of various proteins, including the *N*-linked and *O*-linked glycosylation of glycoproteins [60]. It has been evidenced that *O*-glycans and *N*-glycans function as Golgi export signals to the *trans*-Golgi to promote the constitutive exocytic trafficking of secretory cargoes [61]. Mature and fully processed proteins exit from the TGN which serves as a sorting hub to address them to their proper destination [62]. Thus, tight regulation of trafficking between the ER and Golgi compartments is crucial on the one hand, for the processing and PTMs of proteins from the secretory pathway, and on the other hand, for the proper localization of ER- and Golgi-resident proteins [63,64,65,66]. Indeed, defects in intra-Golgi transport of enzymes implicated in glycosylation strongly affect the maturation, processing and trafficking of glycoconjugates, leading to severe pathologies such as congenital disorders of glycosylation (CDG) [67]. The early steps of the canonical protein secretion pathway are governed by COPII and COPI-mediated transports [1,2,3] (Figure 2b,c), that are both sensitive to *O*-GlcNAc homeostasis.

### 3.1. COPII-Mediated Anterograde Transport

Newly synthesized proteins are exported from the ER by COPII-coated vesicles to the Golgi apparatus. COPII vesicles are composed of two layers: Sar1 and Sec23/Sec24 proteins coat the inner layer where Sec24 interacts with the cargo, and Sec13/Sec31 heterodimers coat the outer layer [68,69]. Near the ER membrane, Sec16 serves as a scaffold to initiate the assembly of COPII at the endoplasmic reticulum exit sites (ERES) [70]. Then, Sec23-interacting protein (Sec23IP) (also known as p125A) is required to promote the displacement of Sec16 from COPII inner layer, which links the two coat layers [71,72]. These Sec proteins are substrates of OGT (Table 1) (Figure 2b). In addition, Sec24B/C, Sec31A and Sec23IP have been identified as O-GlcNAcylated nascent proteins [31].

Functional assays using protein reporters demonstrated that COPII-dependent anterograde transport is sensitive to O-GlcNAc cycling in mammalian cells. OGT inhibition reduced the trafficking of the secreted soluble horseradish peroxidase (ssHRP) [73] and the temperature-sensitive vesicular stomatitis virus G glycoprotein (ts-VSVG-GFP), concomitantly to a decrease in COPII-labeled structures [74]. However, conflicting results on ts-VSVG-GFP transport were obtained in cells treated with Thiamet G, a potent inhibitor of OGA. Cox and colleagues observed a delay in the ER-to-Golgi transport of ts-VSVG in stably transfected COS7 cells, while Cho et al. reported an acceleration of the transport of the glycoprotein from the ER to the plasma membrane in transiently transfected HeLa cells, concomitantly with an increase in COPII-labeled ERES. These conflicting results may be attributed to the differences in cell type, the transfection method, and the time course and concentration of Thiamet G used in their experiment design [73,74]. Furthermore, perturbation in O-GlcNAc cycling affects the cytosolic and membrane-bound distribution of the COPII subunits Sec23A and Sec31A, indicating that O-GlcNAc dynamics regulates the recruitment of COPII subunits at the ERES [73,74].

#### 3.1.1. Sec23A Inner Coat Protein

**Figure 3:**
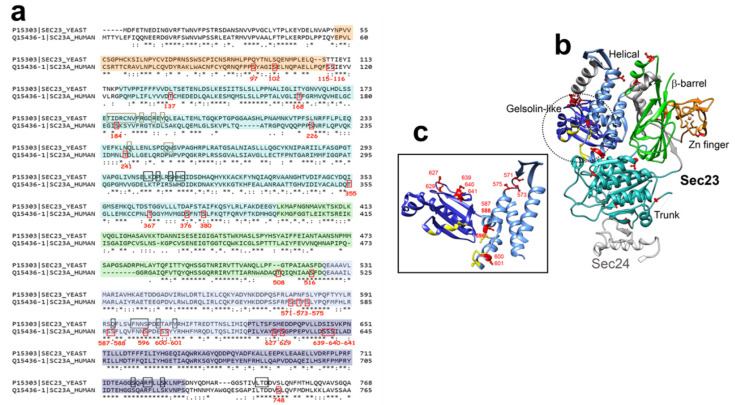
(**a**), Pairwise sequence alignment of yeast and human Sec23a using EMBOSS Needle (http://emboss.open-bio.org/). The secondary structure of human Sec23a is based on sequence homology with yeast Sec23a, according to [75], and is colored as follows: orange, Zinc-finger domain; cyan, trunk domain; green, β-barrel domain; light blue, helical domain; dark blue, gelsolin-like domain. *O*-GlcNAcylated residues are outlined in red. Residues interacting with Sar1 are outlined in black and the ones interacting with Sec24 are outlined in brown (according to [75]). (**b**), Ribbon representation of human Sec23a structure (PDB accession number, 3EGD) using Chimera software (version 1.5). Structural domains are colored as in (**a**). *O*-GlcNAcylated sites are shown in red, residues interacting with Sar1 are in yellow (by sequence homology, according to [75]). Human Sec24 (from residue Glu560 to residue Ala618) is shown in light grey (PDB accession number, 3EGD). (**c**), Enlarged picture of ribbon representation of helical and gelsolin-like domains of Sec23a, colored as in (**b**).

Regarding Sec23A, thirty *O*-GlcNAc sites have been identified so far along the protein sequence: in the Zn-finger domain (S97, S102), the trunk domain (S115, S116, T137, T168, S184, S226, T241, T355, T367, S376, T379, S380), the β-barrel domain (T508, S516), the helical domain (S571, T573, S575, S587, S588, S596, S600, S601), the gelsolin-like domain (S627, S629, S639, S640, S641) and the C-terminal tail (S748) (Table 1) (Figure 3a,b) [73]. Although S184 is localized near the domain of interaction with Sec24, Cox and coworkers showed that its mutation in alanine does not impair their binding (Figure 3b) [73]. The *O*-GlcNAcylation of other residues located near the region of this interaction, such as T168 and S226, could modulate the binding between both subunits (Figure 3b). Furthermore, *O*-GlcNAc modification of S596, S600 and S601 might modulate the binding of Sec23 to Sar1 due to their location close to the region of interaction of both proteins [75] (Figure 3c). Interestingly, *O*-GlcNAcylation at S184 is crucial for proper trafficking of collagen in cultured cells and in drosophila [73]. *O*-GlcNAcylation of Sec23A might alter its interaction with Transport and Golgi organization protein 1 homolog (TANGO1). TANGO1 is an integral membrane protein localized at the ERES. It interacts with Sec23A and Sec16 with its cytoplasmic and C-terminal proline-rich domain (PRD) [71]. TANGO1 mediates export of bulky cargoes from the ER, including collagen, by promoting the recruitment of COPII machinery to form large tubular carriers [76]. PRD motifs of TANGO1 binds to the gelsolin-like domain of Sec23A [77], which contains five *O*-GlcNAc sites (Figure 3). Further investigation is required on whether *O*-GlcNAcylation of these residues modulates the interaction between Sec23A and TANGO1. It should be noted that TANGO1 has been proposed to be *O*-GlcNAcylated, but the identified HexNAc-modified amino acids are located in the ER luminal domain of the protein [76,78], suggesting that TANGO1 could be modified by a unique GalNAc monosaccharide instead of a GlcNAc.

#### 3.1.2. Sec24 Inner Coat Protein

At the inner coat of COPII, Sec24 interacts with the cargo. Human paralogs of Sec24 (A/B/C/D) differ in their affinity for sorting motifs onto cargo proteins [69,79]. Among the *O*-GlcNAc sites mapped on Sec24C, T775 is the only conserved residue in the human paralogs of SEC24, suggesting that *O*-GlcNAcylation of this residue might regulate the whole COPII-dependent trafficking, whereas *O*-GlcNAc cycling on other sites might impact only the anterograde transport of specific cargoes [80]. It is worthy to note that the *O*-GlcNAc site T775 is adjacent to S773 and T776 which are both *O*-GlcNAcylated and phosphorylated [80,81]. Interestingly, global *O*-GlcNAcylation of Sec24C is reduced during mitosis whereas its phosphorylation status is increased [82]. This suggests that, depending on stimuli, a direct competition between phosphorylation and *O*-GlcNAcylation might occur on some residues of Sec24C to finely tune the anterograde transport during cell cycle progression [83]. Changes in *O*-GlcNAc site occupancy induced during cell cycle have also been reported for Sec24A (T160 and T162), Sec24B (T292, T310, T341), Sec16A (T823) and Sec23IP (T118) [30], suggesting that *O*-GlcNAc cycling may be part of the regulatory molecular mechanisms that govern COPII assembly in proliferating cells [84].

#### 3.1.3. Sec31A Outer Coat Protein

Like Sec23A, Sec31A is extensively *O*-GlcNAcylated, with 34 sites identified in human cells (Table 1). Two *O*-GlcNAcylated sites, S269 and S278, are located in the N-terminal WD repeat domain of Sec31A involved in protein–protein interactions, whereas others, such as T903 and S904, are located in disordered regions, or at the C-terminal region of Sec31A. *O*-GlcNAcylation at S964 is involved in the formation of COPII vesicles at the ERES and in the trafficking of ts-VSVG [74], whereas *O*-GlcNAcylation at S1202 is required for the interaction with Sec13 [80]. Moreover, *O*-GlcNAcylation of Sec31A is sensitive to calcium homeostasis in HeLa cells. Depletion of intracellular Ca^2+^ pool by EGTA treatment increases the interaction of Sec31A with OGT and its subsequent *O*-GlcNAcylation, but decreases the interaction of Sec31A with the calcium-binding protein ALG2, which favors the budding of COPII vesicles into the cytosolic space [85]. Conversely, increasing cytosolic Ca^2+^ concentration induces the binding of ALG2 with Sec31A, reduces the *O*-GlcNAcylation of Sec31A and the formation of COPII vesicles at the ERES [85]. Interestingly, phosphorylation of Sec31 by CK2 reduces its fraction bound to ER membrane and the interaction with Sec23 [19]. These results strongly suggest that phosphorylation and *O*-GlcNAcylation of Sec31A may cooperate to regulate the formation of COPII vesicles at the ERES.

Furthermore, *O*-GlcNAcylation of Sec31A is dependent on glucose availability in stably expressing Sec31A HEK293T cells. High glucose levels decrease Sec31A *O*-GlcNAcylation compared to a lower glucose concentration, while the steady-state level of Sec31A remains unchanged [80]. Interestingly, COPII subunits are also regulated at a transcriptional level in response to nutrient availability fluctuations [86,87]. In particular, glucose shortage (0.1/1 mM) increases the mRNA levels of several COPII-coat Sec proteins in human bronchial epithelial cells. In this case, COPII upregulation is needed to counterbalance the loss of EGFR at the cell surface in this scarce nutritional condition [87]. It would be interesting to determine whether changes in *O*-GlcNAc levels onto COPII-Sec components are at the forefront of the molecular mechanisms to rapidly adjust COPII anterograde transport in response to nutrient excess or shortage, before the induction of a more comprehensive response through transcriptional mechanisms.

### 3.2. COPI-Dependent Retrograde Transport

Formation of COPI vesicles begins with the activation of the small GTPase Arf1 which recruits cytosolic COPI at the Golgi membrane (Figure 2c). COPI-coated vesicles are composed of seven core subunits organized in two subcomplexes: the α/β′/ε trimeric complex and the β/γ/δ/ζ tetrameric complex [1,88]. OGT interacts with COP-ε [89] and COP-*α*, -γ, -δ proteins were shown to be O-GlcNAcylated (Table 1) (Figure 2c). In addition, COP-α is O-GlcNAcylated in a co-translational manner [31].

Cox and collaborators showed that disruption of the secretory pathway by Brefeldin A decreases O-GlcNAcylation of COPγ1. COPγ is O-GlcNAcylated at T552 and S554 which are located within the interface of COPγ/COPβ. This suggests that O-GlcNAcylation of COPγ may regulate protein–protein interactions, and subsequently, the COPI-mediated retrograde trafficking [90]. In addition, direct competition with phosphorylation may occur on COPγ1, since five sites (S356, S554, T718, T723, and S725), candidates for O-GlcNAcylation, are also phosphorylated [21,90]. Although functional studies are needed, these studies suggest that OGT could regulate retrograde trafficking by modulating the assembly of COPI subcomplexes.

### 3.3. Arf-GAP and Arf-GEF

Arf small GTPases take part in the intracellular trafficking by recruiting coat proteins onto budding vesicles along the secretory pathway [1,10,91]. Arf proteins cycle between their active-GTP-bound to their inactive-GDP-bound conformations thanks to the antagonist activity of guanine nucleotide-exchange factors (GEFs) and Arf GTPase-activating proteins (ArfGAPs) (Figure 2a). GEFs mediate the exchange of GDP to GTP, inducing conformational changes that allow the insertion of the myristoylated N-terminal helix of Arf into the membranes, hence the recruitment of adaptor proteins and effectors [9,10]. The Golgi-associated BFA-resistant GEF1 (GBF1) functions mostly with Arf1 and mediates the recruitment of the COPI complex to the cis-Golgi [10]. GBF1 is O-GlcNAcylated but the role of the sugar on GBF1 function is fully unknown (Table 1). This PTM could regulate the binding of GBF1 to Arf1, as recently demonstrated for its phosphorylation by the tyrosine kinase Src which induces the subsequent relocalization of polypeptide GalNAc transferases (GALNTs) from the Golgi to the ER [22].

On the other hand, hydrolysis of GTP-bound Arfs is mediated by ArfGAPs (Figure 2a). A few O-GlcNAc sites have been identified on human ArfGAP1 and ArfGAP3 whose activity on Arf1 is involved in COPI vesicle formation [92,93] (Table 1). Furthermore, other ArfGAP superfamily members have been reported to be modified by OGT (Table 1), such as ASAP1 (ArfGAP with SH3 domain, ANK repeat and pleckstrin homology (PH) domain-containing protein 1) which has a GAP activity on Arf1 and Arf5. This is also the case for ACAP1 (Arf-GAP with coiled-coil, ANK repeat and PH domain-containing protein 1) which regulates the activation of Arf6 [94]. Arf6 is involved in the trafficking of recycling endosomes to the plasma membrane and plays a key role in regulating cell adhesion and migration [91,95]. Further studies are needed to determine whether the O-GlcNAcylation of Arf-GAPs is involved in the intracellular trafficking of endosomal compartments.

## 4. Clathrin-Mediated Vesicle Trafficking

Clathrin-coated vesicles (CCVs) are composed of clathrin heavy chains (CHCs) tightly associated with clathrin light chains (LCs) that form the polyhedral coat of the vesicles, together with adaptor protein (AP) complex and endocytic accessory proteins (EAPs). CCVs are involved in the transport of newly biosynthesized proteins from TGN and endosomes, and in the recycling pathway of plasma membrane proteins to the endosomal system [23,96,97].

### 4.1. O-GlcNAcylation of Clathrin and Endocytic Accessory Proteins

Clathrin light chain B (LCB) is *O*-GlcNAcylated in its C-terminal region, at S217/S221 [98], while Clathrin heavy chain 1 (CHC1) is *O*-GlcNAcylated at S97 which is located within a clathrin box motif (CMB) involved in AP2 binding [99] (Table 2). However, to date, the functional relevance of *O*-GlcNAc PTM on clathrin chains is fully unknown.

The formation of CCVs is initiated by the recognition of the cargo by APs which then recruit clathrin to stabilize the curvature of vesicles [23]. AP complexes are heterotetramers consisting of two large subunits (α, δ, γ, ε or ζ), one medium subunit (µ), and one small subunit (σ). AP complexes are crucial for the regulation of CCVs initiation by triggering clathrin assembly and recruiting accessory proteins. AP2 is required for endocytosis at the plasma membrane, while AP1 and AP3 are required for sorting and trafficking of cargoes at the TGN and endosomes [23,62,99]. In addition to the *O*-GlcNAcylation of AP3 which was characterized two decades ago [100], several AP1 and AP2 subunits have been identified as *O*-GlcNAcylated proteins in mammalian cells (Table 2). However, the role of *O*-GlcNAc on AP complexes remains to be investigated.

A variety of EAPs are recruited to coordinate the coat assembly and vesicle formation in clathrin-mediated endocytosis (CME) [23,101]. *O*-GlcNAc sites have been identified on many EAPs, such as AP2-associated protein kinase 1 (AAK1), Epsin 1, Epsin 4, clathrin coat assembly protein AP180 (also known as SNAP91), and phosphatidylinositol-binding clathrin assembly protein M (also known as clathrin assembly lymphoid myeloid leukemia or CALM) (Table 2). The *O*-GlcNAcylation sites of human AP180 are located within its clathrin and adapter (CLAP) domain. A decrease in *O*-GlcNAc modified AP180 was observed in AD brain extracts, concomitantly to a reduction in the level of the protein [102]. The authors suggested that *O*-GlcNAcylation of AP180 regulates its stability but it remains to be experimentally determined. Some of the *O*-GlcNAc sites of CALM are modified in a cell cycle-dependent manner (T356, T363, S364, S453) [30], some of them were shown to be also phosphorylated (S359, S453, T573) (Table 2). The latter suggests that a crosstalk between both PTMs may occur on these residues to finely regulate CALM biological role. Finally, dynamin (Dyn) is involved in the early steps of maturation of vesicles and in the membrane fission of CCVs [23]. *O*-GlcNAcylation of Dyn1 and Dyn2 has been evidenced at least in human cells, while *O*-GlcNAcylation of Dyn3 has been reported only in mouse placenta (Table 2) [103]. For Dyn1, the only *O*-GlcNAc site identified so far is T684 which is located in its coiled-coiled GTPase effector domain.

Palin and colleagues explored the *O*-GlcNAcome of placentas from diabetic mothers. They showed that many proteins involved in CME, such as CHC, AP2A2, and Dyn2 are abnormally *O*-GlcNAcylated in diabetic tissue compared to the control groups [98]. Thus, in addition to phosphorylation-dependent signaling pathways that are crucial to adapt the endocytosis rate to the nutrient uptake and metabolic status of the cell [20], a role for the nutrient-sensing *O*-GlcNAc modification in the regulation of clathrin-dependent endocytic traffic has emerged.

### 4.2. Clathrin-Coated Pits Formation and Transmembrane Receptors Endocytosis

**Figure 4:**
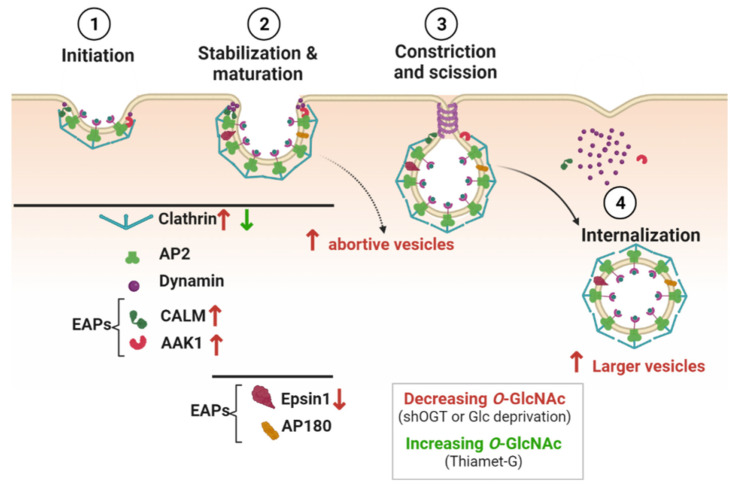
*O*-GlcNAcylation regulates clathrin-mediated endocytosis. Proteins involved in the successive steps of CME and known to be *O*-GlcNAcylated are shown in the figure. Decreasing *O*-GlcNAc levels affects the recruitment of clathrin, CALM, AAK1, and Epsin1 to CCPs (red arrows), and induces more abortive and larger vesicles. Increasing *O*-GlcNAc levels decreases the recruitment of clathrin to CCPs (green arrow).

Rahmani and colleagues have further highlighted molecular insights into the regulation of CME by *O*-GlcNAc cycling. They showed that loss of OGT increases the recruitment of clathrin to clathrin-coated pits (CCPs). This results in an increase in larger nascent CCPs that have a higher propensity to abort, without affecting the fraction of persistent CCPs. A decrease in *O*-GlcNAc levels induced by glucose deprivation has similar effects on CCPs. Conversely, an increase in *O*-GlcNAc levels by Thiamet G treatment slightly reduces the recruitment of clathrin to CCPs and the fraction of short time-life CCPs [104] (Figure 4). Interestingly, *O*-GlcNAc cycling can differentially regulate the recruitment of EAPs that are required for CCP initiation and stabilization [23,105]. Loss of OGT increases the recruitment of CALM but decreases the recruitment of Epsin 1 to CCPs [104] (Figure 4). *O*-GlcNAc sites have been identified on CALM and Epsin 1 (Table 2), but further experiments are needed to determine whether their recruitment to CCPs is regulated by their own *O*-GlcNAc status.

OGT silencing also promotes the colocalization of AAK1 with clathrin. The mutation of the mapped and putative *O*-GlcNAc sites on AAK1 (T360, T448, and T441, S447, T507, S519 and S650) enhances the colocalization of AAK1 with clathrin, suggesting that *O*-GlcNAcylation of AAK1 could negatively regulate the recruitment of the kinase to CCPs (Figure 4). Interestingly, the activity of AAK1 is required to increase CCPs initiation when OGT is silenced, but whether the *O*-GlcNAcylation of AAK1 inhibits its kinase activity remains to be confirmed [104]. Furthermore, phosphorylation of AP2µ2 by AAK1 stabilizes the open conformation of AP2 and its subsequent membrane binding [106]. AP2 can directly interact with transmembrane receptors that need to be internalized, notably via the binding of µ2 subunit with specific motifs in the cytoplasmic domain of cargo [107]. In that respect, modulating the recruitment of AAK1 to CCPs by *O*-GlcNAc cycling could regulate the rate of receptors internalization, as shown for the EGF (EGFR) and transferrin (TfR) receptors [104]. Upon OGT silencing in human retinal pigment epithelial cells, EGFR is proportionally increased in the larger clathrin-labeled structures observed at the cell surface, but TfR recruitment is less effective [104]. Consistently with these results, increasing *O*-GlcNAc levels by glucosamine supplementation enhances the rate of TfR endocytosis in human placenta cells [98]. Furthermore, TfR is potentially *O*-GlcNAcylated [98]. Thus, in addition to the *O*-GlcNAc modification of CCP components, the *O*-GlcNAcylation of TfR per se could also regulate the endocytosis of Tf and the uptake of iron into cells. However, it is likely that the impact of *O*-GlcNAc cycling on CME of TfR is cell-type specific since Tf internalization is not sensitive to the perturbation of *O*-GlcNAc homeostasis induced by knockdown of OGT or OGA in HeLa cells [108].

## 5. Effect of *O*-GlcNAc Cycling on Cargo Trafficking

### 5.1. E-cadherin Trafficking

Cell–cell adhesion is tightly controlled by the amount of the major player of adherens junctions, E-cadherin, present at the cell surface [109]. O-GlcNAcylation negatively regulates E-cadherin levels by a transcriptional mechanism. Indeed, the transcriptional repressor Snail 1 is stabilized by O-GlcNAcylation, thus decreasing E-cadherin expression and promoting cell migration [110]. Besides that, O-GlcNAcylation of E-cadherin has been evidenced by biochemical approaches, although no O-GlcNAc sites have been identified so far [111,112,113]. However, O-GlcNAc residues have been characterized on other members of the cadherin superfamily, such as cadherin-2 (N-cadherin, at S691), cadherin 13 (T-cadherin, at S600 and S607), and E-cadherin’s cytoplasmic partners including β-catenin [114,115].

A blockade of the transport of E-cadherin to the plasma membrane is observed in thapsigargin-induced apoptotic cells, concomitantly to the detection of O-glycosylated forms of E-cadherin [111,112]. This is associated with a lower binding of E-cadherin to p120-catenin (p120), resulting in loss of adhesion [111]. Alternatively, independently of its catalytic activity, OGT inhibits the formation of the E-cadherin/p120 complex through its direct binding to p120 [116]. Changes in the formation of E-cadherin/p120 complex by OGT might also arise from O-GlcNAcylation of the p120-interacting protein Scribble on which three O-GlcNAc sites have been identified (T475, S764, and S1140). Indeed, Scribble is required not only for stabilizing E-cadherin/p120 interaction at the cell cortex, but also for targeting internalized E-cadherin to the lysosomes [117]. It would be interesting to determine whether O-GlcNAc cycling on Scribble impacts the endolysosomal trafficking of E-cadherin.

### 5.2. GLUT4 Trafficking

**Figure 5:**
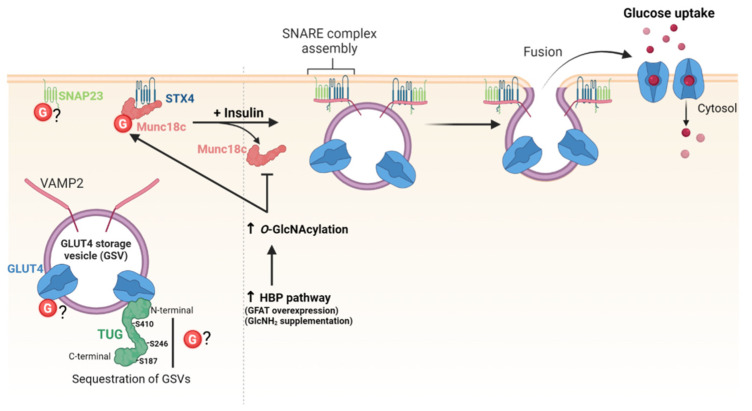
O-GlcNAcylation regulates GLUT4 trafficking and exocytosis.

Regulation of the amount of glucose transporters (GLUTs) at the cell surface is essential to control glucose uptake, especially in insulin-responsive organs. Among GLUTs, GLUT4 is mainly expressed in adipocytes and muscle cells where the regulation of its subcellular localization is important to maintain glucose homeostasis. In the basal state, GLUT4 is retained in TGN, endosomes and in insulin-responsive vesicles, called GLUT4 storage vesicles. Upon insulin stimulation, GLUT4 is released from the storage compartments and transported to the plasma membrane to allow glucose uptake in responsive cells [118]. The docking and fusion of GLUT4 vesicles at the plasma membrane are mediated by the t-SNARE proteins syntaxin 4 (STX4), synaptosomal-associated protein 23 (SNAP23), and the v-SNARE protein vesicle-associated membrane protein 2 (VAMP2). Assembly of the SNARE complex is regulated by the interaction of STX4 with Munc18c (also referred to as syntaxin-binding protein 3). In basal conditions, Munc18c keeps STX4 in an inactive state. Upon insulin stimulation, Munc18c dissociates from STX4 that switches in an open conformation, allowing SNARE complex assembly and exocytosis of GLUT4 [119] (Figure 5).

Enhanced O-GlcNAcylation of GLUT4 and GLUT4-associated proteins was first evidenced by biochemical approaches in mice overexpressing GLUT1 in muscle which fail to stimulate GLUT4-mediated glucose transport in response to insulin [120]. O-GlcNAcylation of GLUT4 has not been confirmed so far, but other studies showed thatO-GlcNAc levels modulate the amount of GLUT4 available at the cell surface by targeting proteins involved in the trafficking of GLUT4-vesicles. First, increasing HBP flux by either glutamine: fructose-6-phosphate amidotransferase (GFAT) overexpression or glucosamine supplementation significantly decreases the amount of GLUT4 in the plasma membrane of rat adipocytes [121]. Later, an underlying O-GlcNAc-dependent molecular mechanism was described, providing a link between chronic elevation of HBP flux, O-GlcNAc levels, and insulin resistance in type II diabetes [122]. O-GlcNAcylation of Munc18c induced by glucosamine supplementation impairs the insulin-stimulated association of STX4 with VAMP2 and the subsequent translocation of GLUT4 at plasma membrane of adipocytes [122] (Figure 5). SNAP23 is also O-GlcNAcylated, but whether it is glycosylated or not in insulin-responsive cells remains to be determined [123,124]. Additionally, tether-containing UBX domain for GLUT4/ASPC1 (TUG) is a tethering protein that sequesters GLUT4-containing vesicles in the cytoplasm in basal conditions. O-GlcNAc sites have been mapped on TUG on S187, S246 and S410, but the functional significance of TUG O-GlcNAcylation is fully unknown [78,125] (Figure 5).

### 5.3. Trafficking of GluA2-Containing AMPARs

Alpha-amino-3-hydroxy-5-methylisoxazol-4-propionate (AMPA)-selective gluta-mate receptor (AMPAR) is a transmembrane receptor for glutamate which regulates synaptic transmission in neurons. AMPAR is a heterotetrameric complex composed by GluA1, GluA2, GluA3 and GluA4. Phosphorylation events at the cytoplasmic C-tail of the subunits modulate AMPAR subcellular localization and function. For example, PKC-dependent phosphorylation of GluA2 at S880 promotes AMPAR clathrin-mediated internalization and depresses synaptic transmission, whereas CaMKII-dependent phosphorylation of murine GluA1 at S831 stimulates the delivery of AMPAR to synapses [126,127]. Besides phosphorylation, a few studies reported that *O*-GlcNAc cycling regulates the subcellular distribution of AMPAR subunits and modulates hippocampal synaptic transmission. Inhibition of OGT by alloxan increases the presence of GluA2 in the plasma membrane fraction from rat hippocampus and enhances AMPAR responses during long-term potentiation. These observations suggest that decreasing *O*-GlcNAc levels stimulates the translocation of GluA2 from the cytosol towards the plasma membrane [128]. Part of the regulatory mechanisms may be due to the *O*-GlcNAc modification of GluA2 through its interaction with OGT [129]. Moreover, elevation of *O*-GlcNAc levels in thiamet G-treated rats disrupts normal hippocampal-dependent learning [129] and decreases the neuronal excitability in rat hippocampus with similar characteristics of GluA2-lacking AMPARs [130]. These works suggest that, as with other PTMs, *O*-GlcNAcylation of GluA2 or associated proteins triggers the trafficking of GluA2-containing AMPARs, and thus modulate the synaptic plasticity through regulation of the cell surface expression of AMPAR [127]. Further investigations are needed to decipher the underlying molecular mechanisms driven by *O*-GlcNAcylation of GluA2.

### 5.4. Amyloid-β Peptide Trafficking

Abnormal deposits of β-amyloid peptides (Aβ) in neurons is one of the major features of Alzheimer’s disease (AD). Aβ are derived from the amyloidogenic proteolytic pathway of the amyloid precursor protein (APP), a ubiquitous protein that is highly expressed in the neuronal system where it plays a key role in synaptic functions and axogenesis [9]. APP is an integral membrane protein that undergoes substantial PTMs, including N-linked and mucin-type O-glycosylations, during its traffic through the secretory pathway. The subcellular localization and trafficking of APP regulate the production of different APP-derived peptides which have either neuroprotective or neurotoxic properties, depending on their proteolytic processing pathway [131]. At the cell surface, APP is cleaved by α-secretase and γ-secretase to generate APP fragments through the so-called non-amyloidogenic pathway. A part of APP is internalized through CME and transported in early endosomes. Then, APP traffics in late endosomes and lysosomes for degradation, goes back to TGN, or is recycled into the plasma membrane. During the intracellular transport of APP, Aβ peptides are generated in the amyloidogenic pathway by the sequential cleavage of APP by β-secretase (also known as BACE1, β-site APP-cleaving enzyme 1) and γ-secretase [131,132].

Enhancing O-GlcNAc levels by the inhibition of OGA reduces Aβ levels and deposit of amyloid plaques in AD mice models [133,134]. T651 and T652 residues have been identified as O-GlcNAc sites in mouse synapses (amino acids conserved in human APP) [41], suggesting that OGT could regulate APP proteolytic processing. Based on site-directed mutagenesis, O-GlcNAcylation of APP at T576 has been proposed to regulate endocytosis and trafficking of APP, therefore decreasing the production of Aβ peptides [135,136]. However, mass spectrometry analyses unambiguously evidenced that T576, T651 and T652 are modified with short O-GalNAc mucin-type glycans rather than with O-GlcNAc monosaccharide [137,138,139]. Downregulation of Aβ levels in high O-GlcNAc conditions could rather result from the O-GlcNAcylation of the nicrastin accessory subunit of γ-secretase at S708 that suppresses its proteolytic activity towards APP [134].

### 5.5. Trafficking of Hyaluronan Synthases

Hyaluronic acid (HA) is a linear polysaccharide belonging to the glycosaminoglycan (GAG) family, composed of repeat units of D-glucuronic acid (GlcUA) and GlcNAc. HA is a major constituent of the extracellular matrix where it plays critical functions in cell proliferation and migration. In contrast to other GAGs which are synthesized in the Golgi apparatus, HA is mainly produced at the plasma membrane by three membrane-bound HA synthases (HAS1-3) which use cytoplasmic UDP-GlcUA and UDP-GlcNAc as donor substrates [140,141]. Thus, localization of HAS at the plasma membrane is one of the mechanisms that regulates HA biosynthesis and content in normal and pathological conditions. HAS are transported to the cell surface by the canonical secretory pathway. At plasma membrane, HAS are endocytosed and recycled back to the cell surface, or degraded in lysosomes [140,141].

HAS2 and HAS3 are both O-GlcNAcylated [142,143]. O-GlcNAcylation of HAS2 at S221 does not interfere with HAS2 traffic, but increases its stability, possibly by competing with phosphorylation at the same residue [142,143,144]. Additionally, O-GlcNAcylation regulates HAS2 expression at a transcriptional level through the O-GlcNAcylation of the transcription factors Sp1 and YY1 by altering their binding to HAS2 promoter [145]. In contrast to HAS2, O-GlcNAcylation regulates the trafficking of HAS3. Elevated O-GlcNAc levels enhance the retention of HAS3 at plasma membrane by reducing its endocytosis rate and slow down the degradation of HAS3 in lysosomes, thus favoring HA synthesis. Conversely, reducing O-GlcNAcylation results in decreased plasma membrane half-life of HAS3, due to the accumulation of the enzyme in enlarged endosomes [143]. In addition, as discussed later in the Section 7, OGT activity and O-GlcNAc levels correlate with the release of HAS3 in extracellular vesicles [143]. Mapping of HAS3 O-GlcNAc sites will help in examining whether the O-GlcNAc-dependent regulation of HAS3 traffic is related to its direct modification, or indirectly by the O-GlcNAcylation of actors of the endolysosomal pathway.

### 5.6. Megalin-Mediated Albumin Endocytosis

Reabsorption of albumin in kidney is important to maintain the homeostasis of the circulating plasma albumin. This process occurs by receptor-mediated endocytosis through the binding of albumin to multiligand transmembrane receptors, such as megalin which is present at CCPs of proximal tubule epithelial cells [146]. The amount of megalin in plasma membrane is positively regulated by PI3K/Akt signaling pathway whose activation is required for fast recycling and cell surface expression of megalin [147]. The trafficking and recycling of megalin are controlled by clathrin and AP1 in MDCK cells [148].

*O*-GlcNAcylation regulates albumin endocytosis. In renal tubular epithelial cells, increasing *O*-GlcNAc levels with high glucose concentration leads to *O*-GlcNAcylation of Akt, resulting in the inhibition of the kinase. This induces a decrease in the expression of cell surface megalin and, consequently, a decrease in albumin endocytosis [149]. Further works are pending to determine whether *O*-GlcNAcylation of clathrin and AP1 subunits participate in the regulation of megalin-mediated albumin endocytosis in proximal tubule cells. Furthermore, a chronic increase in *O*-GlcNAcylation in diabetes might favor the *O*-GlcNAc-driven impaired albumin reabsorption by renal tubular cells, thus contributing to albuminuria in diabetic nephropathy. However, clathrin-independent mechanisms such as caveolae-mediated endocytosis, may also contribute to albuminuria [150,151].

### 5.7. O-GlcNAcylation of HGS Controls the Endosomal Sorting of Internalized Membrane Receptors

**Figure 6:**
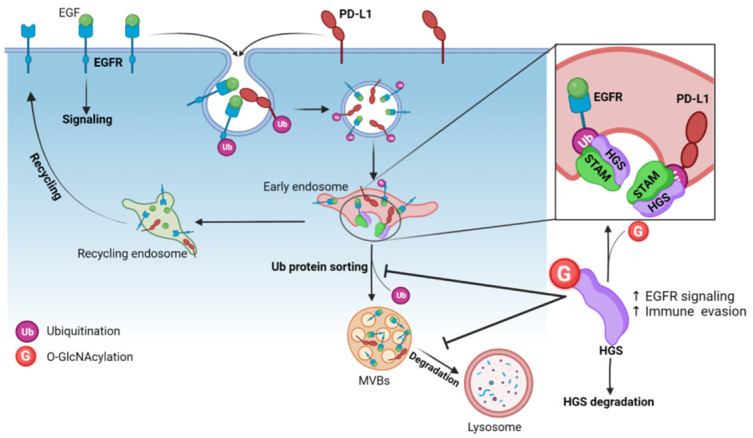
*O*-GlcNAcylation of the ESCRT-0 subunit HGS inhibits the intraluminal sorting of ubiquitinated membrane proteins into MVBs and reduces their lysosomal degradation. Ubiquitination of cell surface EGFR and PD-L1 are internalized in endosomes where they are recognized by the ubiquitin-interacting motifs of HGS, which triggers their sorting into MVBs and degradation into lysosomes. *O*-GlcNAcylation of HGS (at S297/S299/S300) leads to its ubiquitination and prevents the formation of the ESCRT-0 complex HGS-STAM at the endosomal membrane. This leads to accumulation of EGFR and PD-L1 in the endosomal compartments and to their sustained expression in cells.

Degradation of membrane proteins requires the assembly of the endosomal sorting complex required for transport (ESCRT), a highly conserved multi-subunit machinery. ESCRT complexes sequentially assemble on endosomes to generate MVBs and deliver ubiquitinated membrane proteins to lysosomes for degradation. The ESCRT-0 complex is involved in the initial recognition of ubiquitinated membrane proteins in early endosomes. ESCRT-0 is a heterodimer of hepatocyte growth factor regulated tyrosine kinase substrate (HGS/HRS) and signal transducing adaptor molecule (STAM). HGS is also critical for the recruitment of ESCRT-I which is responsible for sorting of ubiquitinated proteins into MVBs [152,153]. Five *O*-GlcNAc sites have been mapped on HGS, all located in a disordered region (Table 3). Wu and colleagues showed that HGS is dynamically modified by *O*-GlcNAc in response to nutrient availability (glucose and glutamine), exogenous stimuli (serum and EGF), or oxidative stress [154]. To date, *O*-GlcNAcylation of HGS has been shown to regulate the lysosomal degradation of EGFR and programmed-death ligand 1 (PD-L1) [154,155] (Figure 6).

#### 5.7.1. Endosomal Sorting of EGFR

Once activated by their ligand, EGFR are internalized into CCVs and transported to early endosomes where they are sorted to be either recycled back to the plasma membrane if they are non-ubiquitinated, or targeted for lysosomal degradation if ubiquitinated [156]. Ubiquitinated EGFR are included into intraluminal vesicles (ILVs) encapsulated in MVBs. Among the *O*-GlcNAcylated residues of HGS (Table 3), the triple mutation S297A/S299A/S300A (3SA) abolishes its *O*-GlcNAcylation and accelerates EGFR degradation [154]. *O*-GlcNAcylation of HGS leads to its ubiquitination, preventing the binding of HGS to STAM, and consequently to the formation of ESCRT-0. *O*-GlcNAcylation of HGS at S297, S299 and S300 also inhibits HGS-EGFR interaction. This leads to the retention of EGFR in early endosomes and inhibits the transport of the receptor to lysosomes, thereby prolonging EGFR-mediated signaling [154] (Figure 6). In agreement with these results, the delivery of a wild-type HGS—cell-penetrating peptide (CPP-G1) into cancer cells competes with the cellular HGS for its *O*-GlcNAcylation and reduces the expression of EGFR. In contrast, incubation of cells with the 3SA-HGS-CPP (CPP-SA) has no effect neither on *O*-GlcNAcylation of HGS, nor on EGFR expression [155]. Wu and colleagues further demonstrated that *O*-GlcNAcylation of HGS supports EGF-mediated liver cancer cell proliferation in vitro and tumorigenesis in vivo by maintaining high expression levels of EGFR that transduces key proliferative signals. Indeed, the growth of xenograft tumors generated from stably expressing 3SA-HGS cancer cells is significantly reduced compared to WT-HGS stably expressing cells. In addition, reduced HGS *O*-GlcNAcylation alleviates the chemoresistance of liver carcinoma cells in vitro [154].

#### 5.7.2. Endosomal Sorting of PD-L1

PD-L1 is an immune checkpoint molecule expressed at the plasma membrane of various cell types. Expression of PD-L1 at the cell surface of cancer cells plays a critical role in immune evasion by suppressing T cell activation through the interaction of PD-L1 with the receptor PD1 on T cells. The development of monoclonal antibodies (mAb) blocking PD-L1 or PD1 is a promising therapeutic strategy to restore an antitumoral immune response [157]. A large proportion of surface-expressed PD-L1 is continuously internalized in early endosomes through CME. Then, PD-L1 is sorted in recycling endosomes to recycle back to the cell surface, or in MVBs to be degraded in lysosomes [157]. Zhu and colleagues recently demonstrated that an increase in *O*-GlcNAcylation of HGS impairs its interaction with internalized PD-L1. This induces a sustained PD-L1 expression in cancer cells due to a decrease in its lysosomal degradation (Figure 6). Conversely, OGT inhibition or mutation of the 3 major HGS *O*-GlcNAc sites (S297A/S299A/S300A) downregulates PD-L1 expression through the sorting of intracellular PD-L1 towards the lysosomes [155].

Interestingly, the delivery of CPP-G1 into cancer cells reduces the *O*-GlcNAcylation of endogenous HGS and the expression of PD-L1 in a dose-dependent manner. In contrast, ectopic expression of CPP-SA has no effect. In addition, CPP-G1, but not CPP-SA, significantly enhances the cytotoxic activity of CD8+ T cells in vitro, indicating that promoting PD-L1 degradation by reducing the *O*-GlcNAcylation of HGS may be an efficient way to stimulate an antitumoral immune response, as the authors demonstrated it in vivo. OGT inhibition synergizes with PD-L1 mAb treatment to significantly increase the infiltration of CD8+ T cells in tumors and reduce tumor growth, compared to PD-L1 mAb treatment alone [155].

To conclude, these two recent studies provide new molecular insights into the role of *O*-GlcNAc modification of HGS, a key component of ESCRT-0 complex, on intraluminal sorting and lysosomal-mediated degradation of membrane receptors. They also highlight a novel role of OGT in tumorigenesis and anti-tumoral therapies through the regulation of cell surface turn-over of critical receptors involved in tumoral growth and immune surveillance. Furthermore, it is likely that future works will link the nutrient-driven *O*-GlcNAc PTM to the other steps of the MVB pathway since subunits of ESCRT-I (Vps37a) and ESCRT-III (Chmp1a, Chmp2a/2b, Chmp4c and Chmp5) complexes are *O*-GlcNAcylated (Table 3).

## 6. Clathrin-Independent Endocytosis

### 6.1. Regulation of the Secretion of Galectin 3

Some cargo proteins are internalized by clathrin-independent processes that are still poorly understood compared to CME [4,158]. During the last few years, the recognition of cell surface glycoproteins by lectins of the galectin family has been shown to regulate clathrin-independent endocytosis (CIE) [158,159]. More particularly, interaction of extracellular galectin 3 (Gal-3) with galactoside-containing glycans leads to an increase in β1 integrin and CMHI internalization, but to a decrease in CD59 internalization due to the galectin lattice-dependent cell-surface sequestration [160,161]. A recent study showed that OGT activity indirectly interferes with CIE of specific cargo proteins by targeting Gal-3 [108]. Disruption of O-GlcNAc cycling by knockdown of OGT or OGA decreases the secretion of Gal-3, resulting in an increase in CD59 endocytosis. Moreover, secreted Gal-3 is preferentially non-O-GlcNAcylated compared with intracellular Gal-3; OGT inhibition increases its secretion. This suggests that low O-GlcNAc level is required for the secretory process of the galectin. Lowering O-GlcNAcylation by glucose privation also increases Gal-3 secretion, compared to normal and high glucose culture media in which secreted Gal-3 is drastically reduced [108]. Surprisingly, mutation of four predicted O-GlcNAc sites located in the N-terminal domain of Gal-3 (T84A, S91A, S92A and T104A) downregulates its secretion [108]. Since Gal-3 forms pentameric oligomers through self-association of its N-terminal domain [162], further experiments are needed to determine whether these mutations induce changes in the oligomerization and secretion rate of Gal-3, independently of its O-GlcNAc status. Taken together, these results show that nutrient-sensing O-GlcNAc modification modulates CIE of specific cargo proteins by regulating Gal 3 secretion [108].

### 6.2. Fast Endophilin-Mediated Endocytosis

OGT may regulate other cellular processes involved in CIE, such as fast endophilin-mediated endocytosis (FEME). This process allows rapid internalization of cargo proteins including tyrosine kinase receptors after stimulation by their ligand [158,163]. Endophilins are Bin/Amphiphysin/Rvs (BAR)-domain-containing proteins involved in the formation and scission of endocytic vesicles. Endophilins A are involved in plasma membrane internalization, both in CME and CIE processes [163,164,165]. Endophilins A2 and A3 are modified by OGT (Table 2). Furthermore, endophilins B1 and B2 which are implicated in membrane dynamics of intracellular organelles are also O-GlcNAcylated [166] (Table 2). The role of O-GlcNAc PTM on their endocytic functions is fully unexplored.

### 6.3. Caveolae-Mediated Endocytosis

The caveolae-mediated endocytosis is mediated by little caves called caveolae, resulting from the invagination of plasma membrane. The formation of caveolae is initiated by the binding of ligands to cargo receptors, allowing the recruitment of caveolin andcavin proteins that cover the nascent vesicles, which leads to their maturation and detachment [167]. Caveolin-1 (Cav-1) is a major structural protein of caveolae vesicles but caveolae-independent functions have also been described in the cytoplasm [168]. Proteomic approaches identified Cav-1 as potentially O-GlcNAcylated, but so far O-GlcNAc sites have not been mapped. However, two studies pointed out a link between Cav-1 and O-GlcNAc dynamics in cancer cells. Elevated O-GlcNAcylation in small cell lung cancer cells increases Cav-1 stability, probably by limiting its ubiquitination and its subsequent proteasomal degradation [169]. Conversely, Cav-1 increases OGT and O-GlcNAc levels in hepatic cancer cells through a post-transcriptional mechanism. Cav-1 alleviates the transcription of miR24 which targets the 3′ untranslated regions of the mRNA of OGT by reducing the expression of the transcription factor RUNX 2 [170]. Cav-1-dependent OGT upregulation promotes the metastatic potential of hepatocellular carcinoma. Further exploration is needed to investigate whether the link between O-GlcNAcylation and Cav-1 is dependent or not of the caveolae-mediated endocytosis process.

## 7. Unconventional Secretory Pathways

Extracellular vesicles (EVs) are a heterogeneous group of cell-derived membranous structures encompassing microvesicles (MVs) and exosomes. MVs are generated by the outward budding and fission of plasma membrane, while exosomes are ILVs formed by inward budding of the late endosomal membrane during their maturation into MVBs. The fusion of MVBs with the plasma membrane allows the secretion of exosomes in the extracellular space [7,171]. EVs allow producing cells to communicate with neighboring cells or distant cells by secreting various types of molecules including nucleic acids, proteins and lipids [172]. As discussed below, OGT expression and *O*-GlcNAc cycling are important for EV protein sorting and unconventional secretion.

### 7.1. Placental OGT Expression and Maternal Circulating EVs

**Figure 7:**
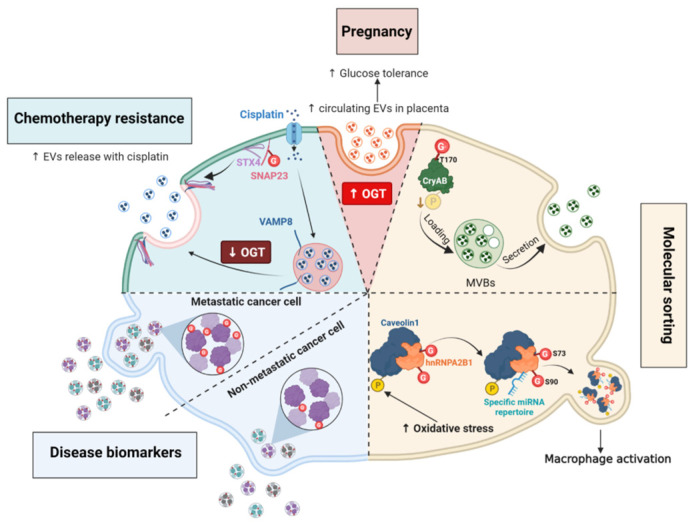
*O*-GlcNAcylation regulates unconventional secretory pathways by regulating molecules sorting and the rate of EV secretion. (i), High placental OGT expression correlates with increases maternal EVs secretion and improvement of glucose tolerance. (ii), Phosphorylation and *O*-GlcNAc modification of CryAB may regulate the encapsulation of the chaperone into MVs in an opposite manner. Secreted CryAB is mainly non-phosphorylated while *O*-GlcNAcylation at T170 promotes its secretion. (iii), The interaction between phosphorylated Cav1 and hnRNPA2B1 induced by oxidative stress promotes the *O*-GlcNAc modification of hnRNPA2B1. *O*-GlcNAcylation at S73 and S90 of hnRNPA2B1 is linked to the binding and release of specific miRNAs that activate macrophages in response to cellular stress. (iv), EVs from metastatic cells have higher levels of *O*-GlcNAcylated encapsulated proteins compared to those contained in EVs from non-metastatic cancer cells. (v), Decreased *O*-GlcNAcylation of SNAP23 facilitates the formation of the SNARE complex SNAP23/STX4/VAMP8 and the secretion of EVs in response to cisplatin treatment.

EV circulation increases during pregnancy. By supporting communication between the mother and the fetus, the production of EVs by the placenta is involved in key physiological processes for fetal development such as angiogenesis, immune modulation and glucose uptake [173]. Maternal stress and gestational diabetes mellitus are associated with changes in concentration, size and content of circulating EVs that may contribute to glucose intolerance and pathophysiological consequences [174,175]. Using genetic targeting of placental OGT, Zierden and colleagues recently showed that high placental OGT expression correlates with an increased concentration of maternal circulating EVs and an improvement of maternal glucose tolerance [175] (Figure 7). Since a significant reduction in maternal glucose sensitivity is known to occur as pregnancy progresses, these results suggest that OGT-dependent increased EV secretion may contribute to improve glucose homeostasis during gestation. A comparative study of EV content between high OGT score and low OGT score-derived placental EVs will help in understanding the underlying molecular mechanisms.

### 7.2. O-GlcNAc Cycling Regulates the Encapsulation of Molecules into Extracellular Vesicles

Molecules encapsulated into EVs help cancer cells to adapt to the tumoral microenvironment [172,176]. Differences in *O*-GlcNAc-modified proteins encapsulated in EVs derived from colorectal cancer cells and metastatic cells have been reported: EVs derived from metastatic cells contain more *O*-GlcNAcylated proteins than EVs from non-metastatic colon cancer cells, in particular the *O*-GlcNAcylated forms of the transitional endoplasmic reticulum ATPase and RuvB-like helicase [177] (Figure 7). The biological significance of this finding is still unexplored, but *O*-GlcNAcylation might regulate EV protein sorting in a direct or indirect manner.

As discussed in the Section 5.5, high *O*-GlcNAc conditions favor HA biosynthesis by regulating the trafficking of HAS3 and promoting its retention at the cell surface [143]. Conversely, high HAS expression and activity increase the secretion of hyaluronan into EVs that are mostly shed from plasma membrane protrusions of various cell types [178]. Elevated *O*-GlcNAcylation increases the release of HAS3-positive EVs whereas reduced *O*-GlcNAcylation decreases this process [143]. Whether the *O*-GlcNAcylation of HAS3 is directly involved in the rate of its encapsulation remains to be elucidated. Actually, this was shown for the chaperone α-crystallin B (CryAB), whose secretion in EVs is directly linked to its *O*-GlcNAc status [179]. The mutation of the major *O*-GlcNAc site T170A of CryAB significantly decreases its encapsulation into exosomes [179]. Moreover, CryAB secreted into exosomes is mainly non-phosphorylated, suggesting that a crosstalk between *O*-GlcNAc and phosphorylation occurs for the encapsulation of cytosolic proteins into EVs, possibly by modifying their aggregation property [39,40,179] (Figure 7).

*O*-GlcNAc modification of specific proteins can also regulate the secretion of other types of molecules into EVs in response to the extracellular environment. For instance, *O*-GlcNAcylated heterogeneous nuclear ribonucleoprotein (hnRNP) A2B1 is engulfed with selected miRNAs into EVs [180]. Lee and coworkers demonstrated that oxidative stress induces the phosphorylation of Cav-1, that increases the formation of hnRNPA2B1/Cav-1 cytosolic complex. This interaction promotes the *O*-GlcNAcylation of hnRNPA2B1 and the sorting of miRNA-bound hnRNPA2B1/cav1 complexes into MVs. Mutation of two *O*-GlcNAc sites at S73 or S90 located in the RNA-binding region of hnRNPA2B1, decreases the binding of hnRNPA2B1 with miR-17 and miR-93 miRNAs specifically. Thus, *O*-GlcNAcylation of hnRNPA2B1 is essential for the encapsulation of miR-17 and miR-93 into MVs (Figure 7). Moreover, variation of the hnRNPA2B1-bound miRNA repertoire in MVs derived from lung epithelial cells regulates the activation of macrophages in response to cellular stress [180].

To conclude, these studies highlighted a role for *O*-GlcNAc cycling in the encapsulation of specific molecules into EVs and in the rate of secretion of EVs. The uptake of these *O*-GlcNAc-driven specific signals by surrounding recipient cells leads to the modulation of biological responses within the tissue, both in physiological and pathological conditions.

### 7.3. O-GlcNAc Cycling Regulates the Formation of SNARE Complexes Required for Exosome Secretion

Secretion of exosomes in the extracellular space requires the formation of SNARE complexes through interactions between v-SNARES present on the vesicles and t-SNARES found at the plasma membrane, such as SNAP23 [7,181]. To date, only one *O*-GlcNAc site at S116 has been identified on SNAP23 [124]. This residue and two other predicted *O*-GlcNAc sites, T123 and T132 (YinOYang server), are located in the linker domain that encompasses the palmitoylated cysteines required for membrane anchorage of SNAP23 [181]. Downregulation of OGT expression decreases SNAP23 *O*-GlcNAcylation and promotes its interaction with STX4 and the v SNARE VAMP8 in ovarian cancer cells. Increased assembly of the SNARE complex raises the secretion of exosomes, which facilitates drug efflux in cisplatin-treated cells [123]. A reduced *O*-GlcNAcylation of SNAP23 facilitates the secretion of EVs, while its phosphorylation at S95 is critical to promote exosome exocytosis [182]. Further investigations are required to examine whether a competition between phosphorylation and *O*-GlcNAcylation occurs onto the linker domain of SNAP23 to regulate the formation of SNARE complexes and hence exocytosis. Furthermore, elevated OGT and *O*-GlcNAc levels in cancer cells might contribute to chemoresistance through downregulation of exosome secretion [123].

### 7.4. O-GlcNAcylation of SNAP29 Regulates the Formation of Complexes Involved in Autophagy

A similar function of *O*-GlcNAcylation has been described on SNAP29 which is required for membrane fusion between autophagosomes and lysosomes. Elevated *O*-GlcNAcylation of SNAP29 disrupts the formation of the STX17/SNAP29/VAMP8 SNARE complex and induces autophagy blockage by impairing the fusion between autophagosomal and lysosomal compartments. In contrast, *O*-GlcNAcylation-defective SNAP29 mutant (mutation of all four *O*-GlcNAc-modified residues at S2, S61, T130 and S153) facilitates the formation of the SNARE complex and promotes autophagic flux. Similarly, reducing HBP flux and depletion or inhibition of OGT restore this catabolic process [183,184,185]. The involvement of *O*-GlcNAcylation of SNAP29 in the regulation of autophagy in response to glucose availability was confirmed in a type I diabetes rat model. In that case, high levels of *O*-GlcNAcylation of SNAP29 impair the SNARE complex formation and induces the blockade of autophagic flux that worsened myocardial injury in diabetic rats. In contrast, decreasing *O*-GlcNAc levels restores autophagy and significantly rescues the abnormal myocardial structures [184]. Furthermore, in cells treated with arsenic, an environmental pollutant, abnormal *O*-GlcNAcylation of SNAP29 also impairs autophagy, that might participate in the arsenic-induced dysfunction in affected populations [185].

### 7.5. GRASP55 O-GlcNAcylation Regulates the Autophagic Flux under Nutritional Stress Conditions

**Figure 8:**
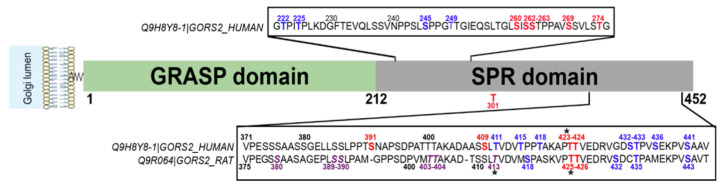
Human GRASP55 is extensively *O*-GlcNAcylated in its C-terminal Ser/Thr rich (SPR) domain. *O*-GlcNAcylated sites are indicated in red and phosphorylation sites in blue. Mutation of the amino acids indicated in purple in the rat sequence decreases the *O*-GlcNAcylation of GRASP55. * denotes phosphorylation and *O*-GlcNAc modification on the same residue.

Golgi reassembly stacking protein 55 (GRASP55/GORASP2) plays a key role in Golgi organization. In concert with GRASP65/GORASP1, GRASP55 forms ordered membrane-associated protein arrays between two opposing membranes via its N-terminal domain [186]. GRASP55 has been proposed to slow down exocytosis to ensure more complete protein glycosylation in the Golgi apparatus and proper sorting at the TGN [187]. GRASP55 is also involved in Golgi-bypassing unconventional secretion of a few cargoes such as cystic fibrosis transmembrane conductance regulator (CFTR) and TGFβ1, especially in cellular stress conditions [8,186]. Ten *O*-GlcNAc sites have been identified in the C-terminal serine/proline-rich (SPR) domain of human GRASP55 (Table 3) (Figure 8). This domain contains also many phosphorylation sites (Figure 8), among which phosphorylation at S441 regulates the oligomerization and ER relocalization of GRASP55 [186,188].

Glucose or amino acid deprivation reduces the *O*-GlcNAcylation of GRASP55 observed in normal nutritional conditions [189]. Lowering GRASP55 *O*-GlcNAcylation enhances its interaction with the autophagosome marker LC3 and induces the binding of GRASP55 to the lysosomal protein LAMP2, both interactions requiring the N-terminal GRASP domain of GRASP55. Bridging LC3 and LAMP2 via GRASP55 facilitates the autophagosome–lysosome fusion, allowing cells to recycle cellular components to counteract nutrient starvation [189]. This study further showed that mutation at S380 increases rat GRASP55 *O*-GlcNAcylation level while mutations at S389, S390, T403, T404 and T413 (which is also a phosphorylation site) reduce its *O*-GlcNAcylation. Simultaneous mutation of all five sites suppresses GRASP55 *O*-GlcNAcylation and enhances the autophagosome maturation into autolysosomes compared to wild-type GRASP55. This indicates that these residues are functionally important for the *O*-GlcNAc-dependent role of GRASP55 in the nutrient-sensing autophagy process [189]. These five residues are conserved in the human GRASP55 sequence (corresponding to S385, S386, T399, T400 and T411), but none of them have been characterized as being *O*-GlcNAcylated until now (Figure 8). Instead, T423 and T424 residues, which are *O*-GlcNAcylated in human cells, are conserved in rodent species (T425 and T426 in mouse and rat GRASP55) (Table 3) (Figure 8). A crosstalk between *O*-GlcNAcylation and phosphorylation might occur at T423 (T425 in rodents) since both PTMs have been characterized on this amino acid, although their functional effect has not been studied so far (Figure 8). Moreover, it remains to be investigated whether the *O*-GlcNAcylation of GRASP55 might regulate the unconventional secretion pathway or the glycosylation process of specific cargoes, under normal and stress conditions [186,187].

## 8. Conclusions

OGT is a nutrient-sensing enzyme and a major player in stress response. Consistent with these functions, and as reviewed here, the homeostasis of *O*-GlcNAcylation modulates intracellular transport in a close relationship with cellular metabolism, particularly with respect to glucose uptake and HBP flux, and stress conditions. Several studies have also highlighted a role of OGT in intercellular communication, in particular by modulating the secretion of encapsulated molecules into MVs. We have just begun to understand the role and physiological relevance of *O*-GlcNAcylation on a few effectors involved in vesicular trafficking. By targeting key proteins involved in the secretory machineries, this PTM can facilitate or prevent the assembly of multiprotein complexes, as evidenced for COP and SNARE complexes. It can also regulate the proteasomal degradation of key actors, as demonstrated for the O-GlcNAcylation of ESCRT-0 subunit HGS, which modulates the turn-over of internalized PD-L1 and EGFR. In the latter case, it is noteworthy that, in turn, EGF-induced phosphorylation of OGT is able to change OGT’s substrate specificity or activity [35]. This reinforces the idea that there may be a complex interplay of PTMs, including *O*-GlcNAc, to adapt protein trafficking and cellular behaviour in response to growth signals and nutritional conditions [20,21]. However, the current knowledge on the tight regulation of endocytosis and exocytosis mechanisms by *O*-GlcNAcylation remains largely incomplete. It appears that the effect of *O*-GlcNAc cycling impairment on intracellular trafficking is dependent on the cargo, the target protein of OGT, and the cellular model. It is important to note that increasing or decreasing *O*-GlcNAcylation by manipulating *O*-GlcNAc enzymes or HBP flux may also indirectly modulate the transport and cell surface expression of cargoes. For example, disruption of the *O*-GlcNAc cycle can interfere with their transcription, as shown for E-cadherin, HAS, and GLUT1 [110,145,190], or with crosstalk with signaling pathways that are also sensitive to metabolic cues, such as AMPK and Akt/mTor pathways [49,50].

The two *O*-GlcNAc databases offer a curated, comprehensive and updated resource of *O*-GlcNAcylated proteins and *O*-GlcNAc sites, not only in mammals but also in all studied species [58,59]. These databases are a wealth of information for further functional studies on the role of *O*-GlcNAc cycling and *O*-GlcNAc site-specific functions in intracellular transport. Combined with genetic and cellular approaches to modulate *O*-GlcNAc levels, in-depth analysis of trafficking taking advantage of retention using selective hook (RUSH) strategy or super-resolution microscopy methods, will help to further understand how *O*-GlcNAc coordinates the molecular mechanisms and the spatiotemporal dynamics underlying the accurate regulation of the secretory pathways.

Finally, the activity of OGT is influenced by its subcellular localization. Changes in *O*-GlcNAcome have been reported mainly according to the relative cytoplasmic and nuclear distribution of OGT and OGA, in response to various signals [38,191]. Recruitment of OGT to the plasma membrane was also reported in response to insulin [192,193]. To our knowledge, very little is known about the potential recruitment of the *O*-GlcNAc processing enzymes at crucial vesicular transport hubs within cells, such as the ERES, TGN, endosomes, or MVBs. Using elegant labeling strategies, considerable efforts have been made in recent years to gain information on the OGT and OGA interactomes, eventually combined with the identification of the *O*-GlcNAc proteome [191,194,195,196]. It should also be noted that the oglcnac.org database contains the OGT-Protein Interaction Network (OGT-PIN) [197], emphasizing that OGT interactors are not necessarily OGT substrate proteins [32]. It is very likely that a very small fraction of OGT transiently interacts with the actors of intracellular transport. Therefore, in-depth subcellular fractionation, e.g., endosomal, Golgi or microtubule-enriched fractions, in combination with such state-of-the-art labeling strategies, is likely to provide clues to define the architecture, dynamics, and topology of the OGT-interacting subcellular proteomes involved in trafficking regulation.

## Figures and Tables

**Table 1:** O-GlcNAcylated proteins involved in the early secretory pathway. Phosphorylation sites are in bold, based on PhosphoSitePlus^®^.

UniProt Accession	UniProt Name	Short Name	Name	*O*-GlcNAcylated Residues
COPI				
P53621	COPA_HUMAN	COPA	Coatomer subunit alpha	S489, T821
P35606	COPB2_HUMAN	COPB2	Coatomer subunit beta’	S423, S432
P48444	COPD_HUMAN	COPD	Coatomer subunit delta	S192, T203, T207, S223, S383, S385
Q9Y678	COPG1_HUMAN	COPG1	Coatomer subunit gamma-1	S134, T135, S186, S187, **S356**, S366, S369, S372, S552, **S554**, S697, T705, T708, **T718**, **T723**, **S725**
**COPII assembly**				
O15027	SC16A_HUMAN	Sec16A	Protein transport protein Sec16A	**S589**, T823, S836, S838, S844, T1001, S1022, S1244, S1245, T1980, S2159
Q9Y6Y8	S23IP_HUMAN	Sec23IP	SEC23-interacting protein	S32, T41, S44, S49, S87, S88, S90, T103, T106, S107, S111, T118, T123, T124, S126, S130, S134, S136, S138, T186, S190, S486
Q92734	TFG_HUMAN	TFG	TRK-fused gene protein	S183, S193, T330, T333, S334, T337, S369, S376, T393
**COPII, inner coat**				
Q15436	SC23A_HUMAN	Sec23A	Protein transport protein Sec23A	S97, S102, S115, S116, T137, T168, S184, S226, T241, T355, T367, S376, T379, S380, T508, S516, S571, T573, S575, S587, **S588**, S596, S600, S601, S627, S629, S639, S640, S641, S748
O95486	SC24A_HUMAN	Sec24A	Protein transport protein Sec24A	S156, S157, T160, S162, T165, T168, T169, S175, S176, S314, S961
O95487	SC24B_HUMAN	Sec24B	Protein transport protein Sec24B	S139, S142, S147, S149, S197, T212, S226, S228, T232, S235, S238, S245, S253, S255, S258, T259, T261, S269, T270, T279, S281, T292, S296, S298, S310, S311, S315, T316, S319, T327, **T329**, T332, T341, S342, T344, S347, S660
P53992	SC24C_HUMAN	Sec24C	Protein transport protein Sec24C	S60, S65, S66, S72, T73, S96, S97, S168, S170, S181, S191, T201, S205, T612, T615, T617, S773, T775, T776
O94855	SC24D_HUMAN	Sec24D	Protein transport protein Sec24D	T9, S13, T35, S418, S421, T427
**COPII, outer coat**				
P55735	SEC13_HUMAN	Sec13	Protein SEC13 homolog	S309, T315
O94979	SC31A_HUMAN	Sec31A	Protein transport protein Sec31A	S269, S278, S451, S527, S532, T658, S666, T674, S762, T774, T903, S904, T910, S914, S915, S917, S938, S948, S963, S964, S965, S1041, S1047, S1048, S1050, S1051, T1073, T1187, T1190, T1195, S1196, S1199, T1201, S1202
Q5JRA6	TGO1_HUMAN	TANGO1	Transport and Golgi organization protein 1 homolog	S579, S591, T864, S865, T1093, S1099
**ER-Golgi SNARE**				
O75396	SC22B_HUMAN	SEC22B	Vesicle-trafficking protein SEC22b	**S164**
**Arf-GAP**				
Q8N6T3	ARFG1_HUMAN	ARFGAP1	ADP-ribosylation factor GTPase-activating protein 1	T141, S144
Q9EPJ9	ARFG1_MOUSE	ARFGAP1	ADP-ribosylation factor GTPase-activating protein 1	T404
Q99K28	ARFG2_MOUSE	ARFGAP2	ADP-ribosylation factor GTPase-activating protein 2	T391, S393, S394
Q9NP61	ARFG3_HUMAN	ARFGAP3	ADP-ribosylation factor GTPase-activating protein 3	S266, S509
Q15027	ACAP1_HUMAN	ACAP1	Arf-GAP with coiled-coil, ANK repeat and PH domain-containing protein 1	S258, T306, S345
Q9ULH1	ASAP1_HUMAN	ASAP1	Arf-GAP with SH3 domain, ANK repeat and PH domain-containing protein 1	T808
Q9QWY8	ASAP1_MOUSE	ASAP1	Arf-GAP with SH3 domain, ANK repeat and PH domain-containing protein 1	T823
**Arf-GEF**				
Q92538	GBF1_HUMAN	GBF1	Golgi-specific brefeldin A-resistance guanine nucleotide exchange factor 1	S283, **S1784**, S1856

**Table 2:** *O*-GlcNAcylated proteins involved in clathrin-dependent or -independent endocytosis. Phosphorylation sites are in bold, based on PhosphoSitePlus^®^.

UniProt Accession	UniProt Name	Short Name	Name	*O*-GlcNAcylated Residues
Clathrin chains				
Q00610	CLH1_HUMAN	CHC1	Clathrin heavy chain 1	S97, T1180
P53675	CLH2_HUMAN	CHC2	Clathrin heavy chain 2	not assigned
P09496	CLCA_HUMAN	LCA	Clathrin light chain A	not assigned
P09497	CLCB_HUMAN	LCB	Clathrin light chain B	S217 or S221
**AP complex**				
Q9BXS5	AP1M1_HUMAN	AP1M1	AP-1 complex subunit mu-1	S28
O95782	AP2A1_HUMAN	AP2A1	AP-2 complex subunit alpha-1	T189, S611
P17427	AP2A2_MOUSE	AP2A2	AP-2 complex subunit alpha-2	T126
P63010	AP2B1_HUMAN	AP2B1	AP-2 complex subunit beta	S671, S672
Q9DBG3	AP2B1_MOUSE	AP2B1	AP-2 complex subunit beta	S79, S90
O00203	AP3B1_HUMAN	AP3B1	AP-3 complex subunit beta-1	S671
**Endocytic accessory proteins**				
Q9Y6I3	EPN1_HUMAN	EPN1_HUMAN	Epsin-1	T517, S536
Q14677	EPN4_HUMAN	EPN4, CLINT1	Epsin-4, Clathrin interactor 1	S311, S312, T315, S407, S409, S420, S624
Q99KN9	EPN4_MOUSE	EPN4, CLINT1	Epsin-4, Clathrin interactor 1	S90, T273, S320, S327, S328, S630
Q13492	PICAL_HUMAN	CALM, PICALM	Phosphatidylinositol-binding clathrin assembly protein (Clathrin assembly lymphoid myeloid leukemia)	S248, T352, S353, T355, T356, **S359**, S362, T363, S364, T370, S409, S443, S452, **S453**, S497, T498, T517, S565, **T573**, S576, T585, T586
Q7M6Y3	PICAL_MOUSE	CALM, PICALM	Phosphatidylinositol-binding clathrin assembly protein (Clathrin assembly lymphoid myeloid leukemia)	T301, **T355**, T356, **S359**, S362, T363, S364, T370, S453, T460, S481
Q2M2I8	AAK1L_HUMAN	AAK1	AP2-associated protein kinase 1	T354, T359, T360, S363, T441, T445, S447, T448, T507, S519
Q3UHJ0	AAK1_MOUSE	AAK1	AP2-associated protein kinase 1	T359, T360, S406, S414, S416, T445, T448, S572, T578, S648, T740, T746, T747, S749, S751, T846
O60641	AP180_HUMAN	AP180	Clathrin coat assembly protein AP180 (SNAP91)	T309, T310, T312, S341, T626, T627, S629
Q61548	AP180_MOUSE	AP180	Clathrin coat assembly protein AP180 (SNAP91)	S303, S305, S306, T309, T310, T312, T333, S621, S624, T625
**Dynamin GTPase**				
Q05193	DYN1_HUMAN	Dnm1	Dynamin-1	T684
P39053	DYN1_MOUSE	Dnm1	Dynamin-1	T748, T749
P50570	DYN2_HUMAN	Dnm2	Dynamin-2	not assigned
Q8BZ98	DYN3_MOUSE	Dnm3	Dynamin-3	T769
**Clathrin-independent endocytosis**				
Q99961	SH3G1_HUMAN	Endophilin 2	Endophilin A2	T278/T279, S286
Q62419	SH3G1_MOUSE	Endophilin 2	Endophilin A2	T27, T284
Q99963	SH3G3_HUMAN	Endophilin 3	Endophilin A3	T55
Q9Y371	SHLB1_HUMAN	Endophilin B1	Endophilin B1 (Bax-interacting factor 1, Bif-1)	not assigned
Q9NR46	SHLB2_HUMAN	Endophilin B2	Endophilin B2	not assigned

**Table 3:** *O*-GlcNAcylated proteins involved in unconventional secretory pathways. Phosphorylation sites are in bold, based on PhosphoSitePlus^®^.

UniProt Accession	UniProt Name	Short Name	Name	*O-*GlcNAcylated Residues
MVBs formation and sorting of endosomal cargo proteins into MVBs				
ESCRT-0				
O14964	HGS_HUMAN	HGS	Hepatocyte growth factor-regulated tyrosine kinase substrate	S297, S299, S300, S310, S315
**ESCRT-I**				
Q8NEZ2	VP37A_HUMAN	VPS37A	Vacuolar protein sorting-associated protein 37A	S172, S174, T178
**ESCRT-III**				
Q9HD42	CHM1A_HUMAN	CHMP1A	Charged multivesicular body protein 1a	not assigned
O43633	CHM2A_HUMAN	CHMP2A	Charged multivesicular body protein 2a	not assigned
Q9UQN3	CHM2B_HUMAN	CHMP2B	Charged multivesicular body protein 2b	S80
Q96CF2	CHM4C_HUMAN	CHMP4C	Charged multivesicular body protein 4c	not assigned
Q9NZZ3	CHMP5_HUMAN	CHMP5	Charged multivesicular body protein 5	T18, T23
**Autophagosome Maturation**				
Q9H8Y8	GORS2_HUMAN	GRASP55	Golgi reassembly-stacking protein 2	S260, S262, S263, S269, T274, T301, S391, S409, **T423**, T424
Q99JX3	GORS2_MOUSE	GRASP55	Golgi reassembly-stacking protein 2	**T425**, **T426**
**SNARE Complex**				
O00161	SNP23_HUMAN	SNAP23	Synaptosomal-associated protein 23	S116
O95721	SNP29_HUMAN	SNAP29	Synaptosomal-associated protein 29	S2, S61, T130, S153

## Data Availability

Not applicable.

## Abbreviations

AAK1, adaptor-associated kinase 1; AD, Alzheimer’s disease; AP, adaptor protein; APP, amyloid precursor protein; Arf, ADP-ribosylation factor; Aβ, β-amyloid peptide; CCP, clathrin-coated pit; CCV, clathrin-coated vesicle; CHC, clathrin heavy chain; CIE, clathrin-independent endocytosis; CME, clathrin-mediated endocytosis; COP, coat protein complex; EAP, endocytic accessory protein; ER, endoplasmic reticulum; ERES, endoplasmic reticulum exit site; ESCRT, endosomal sorting complexes required for transport; EV, extracellular vesicle; GAG, glycosaminoglycan; Gal-3, galectin 3; GAP, GTPase-activating protein; GBF1, Golgi-associated BFA-resistant GEF1; GEF, guanine nucleotide-exchange factor; GLUT, glucose transporter; HA, hyaluronic acid; HAS, HA synthase; HBP, hexosamine biosynthetic pathway; HGS, hepatocyte growth factor regulated tyrosine kinase substrate; ILV, intraluminal vesicles; MVB, multivesicular body; OGA, *O*-GlcNAcase; *O*-GlcNAc, *O*-linked-β-N-acetylglucosamine; OGT, *O*-GlcNAc transferase; PD-L1, programmed-death ligand 1; PTM, post-translational modification; Sec23IP, Sec23-interacting protein; SNAP, synaptosomal-associated protein; SNARE, soluble NSF attachment protein receptor; TANGO1, transport and Golgi organization protein 1 homolog; TGN, *trans* Golgi network; VAMP, vesicle-associated membrane protein.
